# Multifocal Intraoral Melanoacanthomas: Two Case Reports and a Literature Review

**DOI:** 10.7759/cureus.26946

**Published:** 2022-07-17

**Authors:** Hawra A Aljanobi, Muneer H Alshuyukh, Manayer S Husain, Waleed M Alqahtani

**Affiliations:** 1 Oral Pathology, Biomedical Dental Science, College of Dentistry, Imam Abdulrahman Bin Faisal University, Dammam, SAU; 2 Biomedical Dental Science, College of Dentistry, Imam Abdulrahman Bin Faisal University, Dammam, SAU; 3 Department of Oral and Maxillofacial Surgery, King Khalid Hospital, Alkharj, SAU

**Keywords:** melanocyte, multifocal oral lesion, diffuse pigmentation, oral pigmented lesion, oral melanoacanthoma

## Abstract

Intraoral melanoacanthoma is a rare, reactive pigmented lesion that is mostly seen in black individuals with a tendency to occur more frequently in younger females. The color of melanoacanthoma may vary from brown to black, and it is commonly seen as a solitary lesion in the buccal mucosa. This lesion requires no treatment, and no malignant potential has been observed to date. The clinical presentation of multifocal oral melanoacanthoma (MOMA) (the rate of growth and the recurrence) is usually diagnostic. On the other hand, solitary oral melanoacanthoma might be difficult to diagnose, and a biopsy should be performed and examined by a well-trained oral pathologist. Here, we report two cases of MOMA with an unusual location in one case.

## Introduction

Intraoral melanoacanthoma is a rare, mixed epithelial pigmented lesion that is mostly seen among blacks with a tendency to occur more frequently in younger females [1]. These lesions are reactive, grow rapidly, and may appear within days or weeks, although no malignant potential has been reported to date [1,2]. The color of the lesion may vary from brown to brown-black to blue-black with a flat or slightly raised surface [1]. Although oral melanoacanthoma (OMA) usually presents as an asymptomatic solitary lesion, in a few case reports, the lesions were accompanied by painful, burning, and itching sensations [1]. The most common site is buccal mucosa, followed by the palate, lips, rarely in the gingiva (5.6%), and tongue (2.8%) [3]. The etiology of such lesions remains unclear; however, it is thought to be due to physical or chemical trauma [4]. Furthermore, the lesion is known to be associated with some systemic diseases such as hyperthyroidism and Addison’s disease [5]. Solitary OMA is indistinguishable from other pigmented oral lesions including malignant oral melanomas; hence, an incisional biopsy is recommended. In most cases, the lesion regresses and disappears after biopsy without a need for treatment [1,2].

Here, we report two cases of multifocal oral melanoacanthoma (MOMA) with an unusual location in one case. In addition, we review and summarize most of the reported cases of MOMA in the literature.

## Case presentation

Case one

A 36-year-old Saudi female patient presented to the Oral and Maxillofacial Surgery Department complaining of black pigmentation on her left and right buccal mucosa. She reported that the lesions had appeared suddenly for two weeks and had been gradually increasing in size. The lesions were not associated with pain or any change in sensation. She also denied any constitutional symptoms, including fever, chills, night sweats, appetite changes, and weight loss. The patient was a non-smoker. In addition, her medical history did not reveal any chronic illness or allergy, and there was no history of medication use. The patient had a history of dental implants and prosthetic crowns on the right and left posterior lower teeth a long time ago. Extraoral examination showed no significant findings.

**Figure 1:**
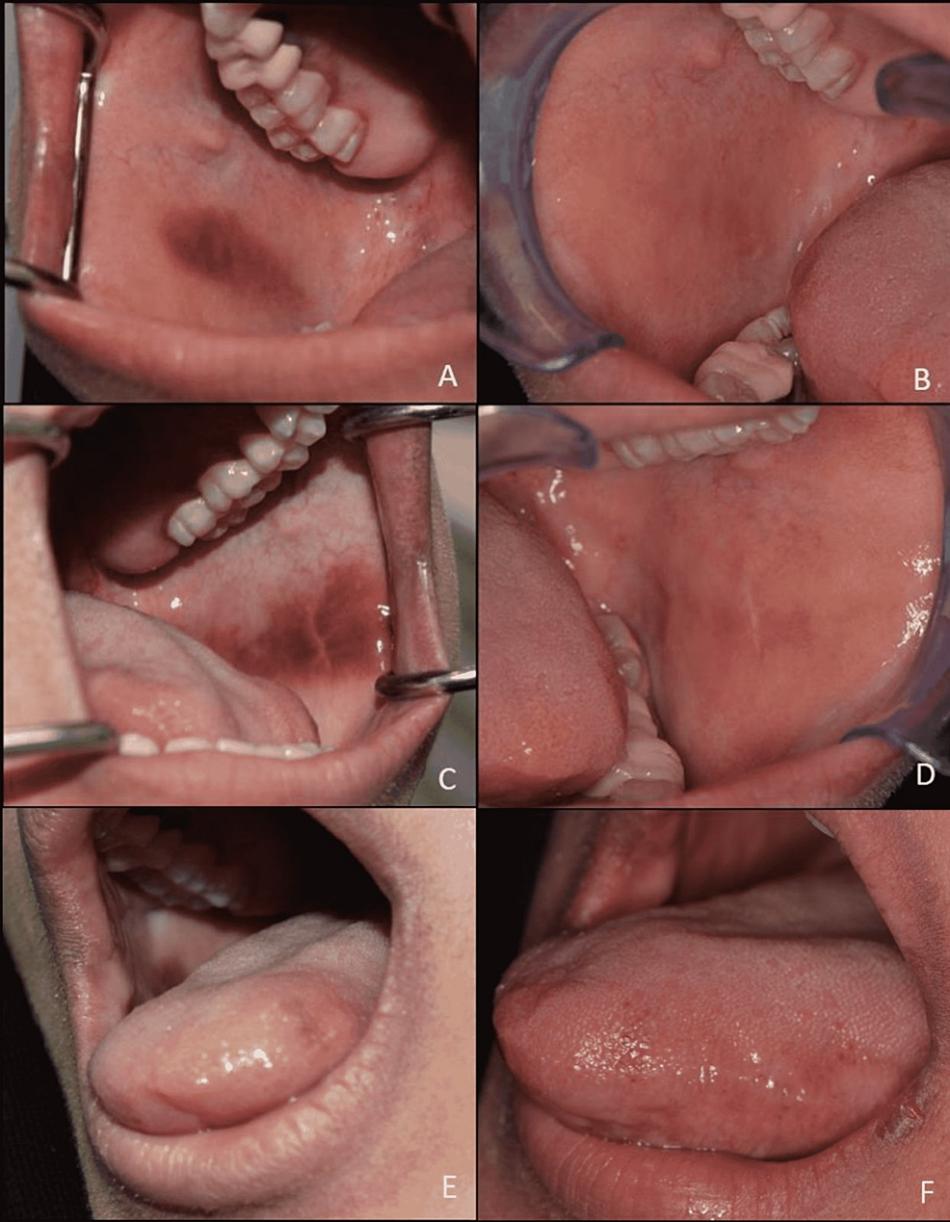
Multifocal melanoacanthomas. Well-demarcated brown-black macule on the right buccal mucosa (A), left buccal mucosa (C), and brown macule on the left lateral side of the tongue (E). Complete resolution of the lesion noted on the right side (B), left side (D), and the tongue (F) after eight months.

Intraoral examination revealed a brown-black pigmented lesion on the left and right buccal mucosa, with the left side being more diffuse (Figure 1). The lesions were slightly raised with a rough texture. No ulceration, active bleeding, or tenderness was detected at the time of examination. An incisional biopsy was taken from the left buccal lesion to rule out any malignancy. The initial histopathological diagnosis by a general pathologist was an acute lichenoid reaction. A second opinion by an oral and maxillofacial pathologist was sought, and the case was diagnosed as OMA.

**Figure 2:**
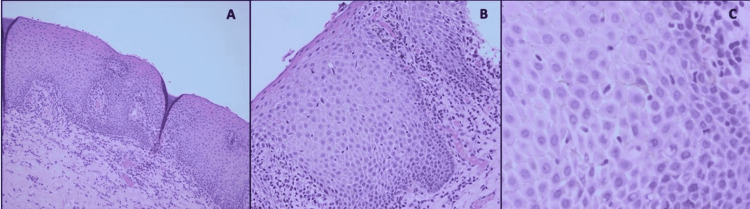
Photomicrograph of hematoxylin and eosin-stained sections. (A) Stratified squamous epithelium with acanthosis, spongiosis, and basal cell layer hyperplasia (20×). (B and C): The presence of numerous dendritic melanocytes that contain brownish pigments in their cytoplasm, extending throughout the entire thickness of the epithelium also shows basal and para-basal melanocytes with melanin incontinence. Chronic inflammatory cell infiltrate can be seen in the superficial lamina propria (40×, 100×).

Histological examination showed a wedge of oral mucosa surfaced by acanthotic and spongiotic stratified squamous epithelium with numerous dendritic melanocytes throughout the thickness of the epithelium. Increased melanosis was seen at the basal and para-basal level with mild chronic inflammatory cell infiltration in the superficial connective tissue (Figure 2).

A two-week follow-up of the patient showed a new lesion on the left lateral side of the tongue with the same color as the buccal lesions but smaller in size (Figure 1). The eight-month follow-up showed complete resolution of all lesions.

Case two

**Figure 3:**
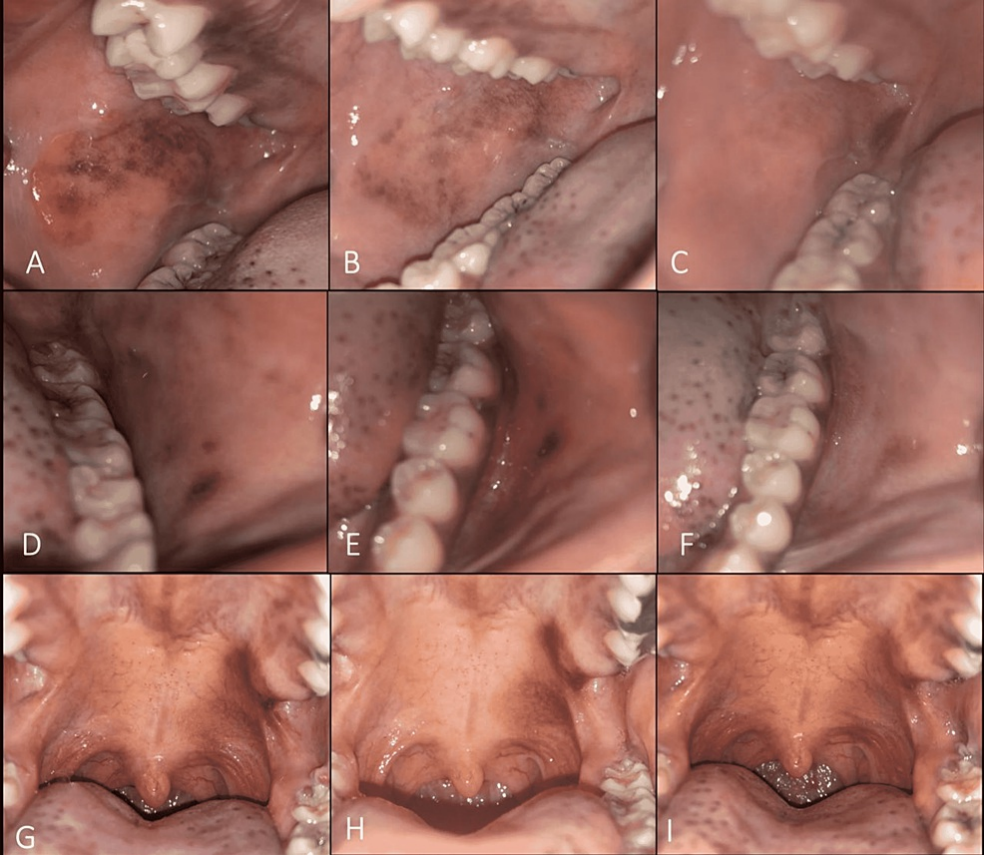
Multifocal melanoacanthomas. Right side buccal mucosa: (A) first presentation, (B) after two months, and (C) after two years. Left side buccal mucosa: (D) first presentation, (E) after two months, and (F) after two years. Unilateral diffuse brownish pigmentation involving the left soft palate: (G) first presentation, (H) after two months, and (I) after two years.

A 21-year-old black Saudi female dental student presented to the Oral Medicine Clinic complaining of rapidly growing brown discolorations on multiple areas in her mouth for three months. The patient was a non-smoker, medically fit, and did not take any medication. The extraoral examination was normal. Intraoral examination revealed multiple, diffuse, smooth brownish-black macules with irregular erythematous border on the right and left buccal mucosa (Figure 3). Furthermore, similar lesions were seen on the left side of her soft palate (Figure 3). She reported that the lesions appeared at variable times. The lesions were completely painless, and no history of trauma was reported. Based on the history, behavior of the lesion, and clinical examination, a diagnosis of OMA was made. At the two-month follow-up, slight resolution of some of the lesions was observed. At the two-year follow-up, complete resolution of most of the lesions was noted.

## Discussion

Mishima and Pinkus were the first to describe melanoacanthoma in 1960 as a benign mixed skin tumor of basal and prickle cell keratinocytes and pigment-laden dendritic melanocytes [6]. The first case of OMA was reported in 1979 [6]. Although OMA is an uncommon oral pigmented condition that commonly presents as a solitary lesion, in a few cases, it can be multifocal.

Most of the previously reported cases of MOMA have been summarized in Table 1. MOMAs are much more common among females than males, with a female-to-male ratio of 3.8:1. It also has a predilection for black ethnicity, with a white-to-black ratio of 1:1.75. Case reports have shown a wide age range, with the youngest reported age of eight and the oldest being 77 years, with an average age of 42 years.

**Table 1:** Summary of previously reported cases of MOMA. M: male; F: female; B: black; W: white; NA: not available; MOMA: multifocal intraoral melanoacanthomas

Author	Year	Number of MOMA	Sex	Race	Age	Location	Clinical presentation	Suggested causative factor/s
Goode et al. [8]	1983	1	F	B	19	Palate, alveolar ridge	Brown macule	Prosthesis
Horlick et al. [9]	1988	2	M, F	B, W	36, 16	Buccal mucosa (bilateral), palate, lower lip	Dark brown macule, brown-black macule	Patients treated for chronic asthma. Sun exposure
Zemtsov et al. [10]	1989	1	F	B	34	NA	NA	NA
Chandler et al. [11]	1997	1	F	B	54	Soft palate, oropharynx	Black macule	Persistent dry cough
Fatahzadeh et al. [2]	2002	1	M	B	39	Buccal mucosa, hard palate	Brown macule	Cheek sucking and biting
Fornatora et al. [12]	2003	3	F, F, F	B, B, B	77, 28, 24	Buccal mucosa (bilateral), retromolar pad	Brown macule	NA
Andrews et al. [7]	2005	1	M	B	45	Ventral tongue, the floor of the mouth	Tender and ulcerated	Acquired immunodeficiency syndrome
Yarom et al. [3]	2007	1	F	W	60	Hard palate and gingiva	Brown macule	NA
Marocchio et al. [13]	2009	1	F	B	74	Buccal mucosa, lips, gingiva, and tongue	Black/Brownish	Prosthesis
Brooks et al. [14]	2009	1	F	W	60	Gingiva, hard palate	Light brown-brown macule	NA
Galindo-Moreno et al. [15]	2011	1	F	W	63	Hard palate, soft palate, maxillary touristy, buccal mucosa	Brown-black macule	Dental implant surgery, iron tablets
Geetha et al. [16]	2011	1	M	B	8	Buccal mucosa (bilateral), gingiva, alveolar mucosa	Blackish-brown macule	NA
Gupta et al. [6]	2012	1	F	B	22	Buccal mucosa (bilateral), hard palate, soft palate, retromolar areas	Diffuse black-brown macule	NA
Stoopler et al. [17]	2017	1	F	W	50	Maxillary and mandibular gingiva	Black macule	NA
Dantas et al. [5]	2017	1	F	B	50	Upper lip mucosa, gingiva, tongue, buccal mucosa	Itchy brown-black macules with whitish plaques	Addison’s disease, Graves’ disease
Tandon et al. [18]	2018	1	F	NA	55	Buccal mucosa (bilateral), upper and lower labial mucosa	Brown-black macule	NA
Gonçalves et al. [19]	2019	1	F	W	53	Gingiva and upper lip	Black-brown macule	NA
Zaki et al. [20]	2019	1	F	W	56	Hard palate, palatal gingiva	Brownish macule	Laugier–Hunziker syndrome
Albagieh et al. [4]	2020	1	F	NA	21	Gingiva, upper and lower labial mucosa	Diffuse black-brown macule	Teeth whitening strips

The lesion usually presents as brown/black macules of variable sizes, which might be raised or elevated. It usually involves two sites rather than being diffuse (three sites and more). The two most commonly involved sites are the hard palate and gingiva, followed by bilateral buccal mucosa. The least documented site to be involved is the floor of the mouth [7]. Some associated symptoms have been reported such as tender and ulcerated, burning sensation, itchiness, and pain [5,7].

The clinical presentation of MOMA (the rate of growth and the recurrence) is usually diagnostic, and a biopsy is rarely needed. On the other hand, solitary OMA might be challenging to diagnose based on clinical presentation only, especially with no history of onsite and recurrences. In such cases, the lesion might be mistaken as malignant melanoma based on the size of the lesion, border irregularity, and rapid growth; hence, a biopsy is indicated. In one of our cases, the diagnosis was made based on histological examination, while, in the other case, the diagnosis was made based on the history of recurrence and presentation of the lesions. However, even with histological examination, the diagnosis can be easily missed if not reviewed by a well-trained practitioner in the field of maxillofacial pathology. Histologically, the lesion has unique features of showing numerous dendritic melanocytes extending between the spinous epithelial cells with acanthosis and spongiosis of the epithelium.

MOMA has been documented in association with local factors such as dental prosthesis [8,13], chronic trauma to the cheeks [2], dental implant surgery [15], and teeth whitening strips [4], as well as some systemic diseases and syndromes such as acquired immune deficiency syndrome [7,11], Laugier-Hunziker syndrome [20], Addison’s disease, and Graves’ disease [5]. In our first case, the patient had a history of dental prosthesis and implant, which might have contributed to the development of melanoacanthoma. In addition, the patient had frequent bruising in her legs, although her lab results showed normal values. In case two, no history of any causative factor was suggested. Moreover, none of our patients were smokers.

Differential diagnosis of MOMA includes drug-induced melanosis, systemic disease (e.g., Addison’s disease), syndromes (e.g., Peutz-Jeghers syndrome and McCune-Albright syndrome), smoker melanosis, and racial pigmentation. Malignant melanoma is usually not included in the differential of MOMA as it develops as a solitary lesion and not as a multifocal lesion.

However, some clinicians may hesitate to finalize the diagnosis based solely on the clinical examination. Recently, dermoscopy has been introduced to oral medicine clinics, allowing proper magnification and visualization of the morphologic features of oral pigmented lesions non-invasively [21]. The procedure helps differentiate between melanotic nevi and malignant melanoma; however, no clear criteria have been presented to validate using dermoscopy to discriminate oral melanoma from melanoacanthoma [21]. More studies should be performed to decrease the morbidity of biopsy, especially concerning MOMA. In general, the ABCDE criteria (Asymmetry, Border, Color, Diameter, Evolution) are used when melanoma is suspected [22]. The solitary malignant melanoma is often asymmetrical with ragged, notched, or blurred borders [22]. The color of the lesion is not uniform with a white, reddish, or blue veil [22]. A diameter of more than 6 mm is usually characteristic of melanoma [22]. Lastly, melanoma often changes its characteristics over time such as size, shape, or color [22].

Melanoma has variable histological presentation which can mimic carcinomas, neuroendocrine tumors, sarcomas, lymphomas, and others [23]. Therefore, immunohistochemical staining is an important aid in diagnosing malignant melanoma. Among the most commonly used markers are human melanoma black-45 (HMB-45), Melan-A, and S100, all of which are positive in malignant melanoma [23]. Although solitary melanoacanthoma might mimic malignant melanoma clinically, they have no histological similarity and can be distinguished effortlessly based on histological presentation.

Once the diagnosis of MOMA is confirmed, no further treatment is required as it is a self-limited process. Argon plasma coagulation has been used in one study to treat MOMA with no recurrence after a one-year follow-up [7]. No malignant transformation has been reported so far [7].

## Conclusions

It is crucial that dental practitioners have sufficient knowledge and approach to diagnose MOMA. This will minimize the number of incision biopsies that have to be obtained from patients to reach a diagnosis. Additionally, it will spare the patient from unnecessary pain and expenditures for a lesion that can be easily identified based on clinical presentation.
